# Scoping Review of the Published Guidelines for the Management of Traumatic Brachial Plexus Injuries

**DOI:** 10.7759/cureus.79266

**Published:** 2025-02-19

**Authors:** Michael A Weekes, Christopher McGhee, Caroline Miller, Abdus Burahee, Ian Rouse, Vaikunthan Rajaratnam, Dominic Power

**Affiliations:** 1 Hands, Plastics, and Peripheral Nerve Research Network, University Hospitals Birmingham NHS Foundation Trust, Birmingham, GBR; 2 Peripheral Nerve Surgery, University Hospitals Birmingham NHS Foundation Trust, Birmingham, GBR; 3 Trauma and Orthopedics, Queen Elizabeth Hospital Birmingham, Birmingham, GBR; 4 Trauma and Orthopedics, University of Puthisastra, Phnom Penh, KHM; 5 Hand Surgery Unit, Orthopedic Surgery, Khoo Tech Puat Hospital, Yishun, SGP

**Keywords:** brachial plexus injury, guideline, management algorithm, peripheral nerve surgeries, ­trauma

## Abstract

A traumatic brachial plexus injury (TBPI) is a rare yet debilitating condition with a typical incidence of 0.6-3.9 per 100000 annually, predominantly affecting young, economically active males following motorcycle accidents. Delayed diagnosis and treatment are associated with poorer functional outcomes, significant individual disability, and societal burdens, including loss of vocational potential and increased care costs. Individuals in resource-limited settings are particularly vulnerable to receiving suboptimal care. This study aimed to identify and evaluate existing published guidelines for the management of TBPI.

A systematic review of literature from nine medical databases, using standardized search methods with results screened for relevance and analyzed using modified Appraisal of Guidelines for Research and Evaluation II-global rating scale (AGREE II-GRS) guidelines to determine quality.

The search identified 1163 papers, of which eight met the inclusion criteria: six original research articles, one national guideline, and one departmental guideline. Six studies included treatment algorithms; however, only two categorized nerve transfers by pathology (upper (C5-6 ± C7), lower (C8-T1), or pan-plexus). None provided a sequenced or ordered approach to surgical management. Outcome reporting was inconsistent across studies. A modified AGREE II-GRS analysis indicated that the guidelines were appropriately targeted to relevant professional and patient groups.

At present, there are no TBPI guidelines with structured, consensus-based recommendations for managing this condition within acute or secondary care settings in low- and middle-income countries (LMICs). Our study identified eight published and accessible guidelines with treatment algorithms, but none provided a comprehensive management regimen covering all aspects of TBPI care. Developing such a guideline for LMICs is challenging due to the rarity, variability, and complexity of this pathology. Any guideline designed for this context must account for the health economics, resource availability, logistical barriers, and personnel constraints required to ensure a fit-for-purpose management plan for a TBPI service.

## Introduction and background

Adult traumatic brachial plexus injury (TBPI) is a challenging clinical problem with profound implications for patients’ quality of life. The complex nature of TBPI care arises from variations in injury severity, timing of referral and intervention, associated injuries, patient pre-morbid status, and method of reconstruction adopted. Factors such as access to healthcare resources and patient engagement with rehabilitation also critically influence outcomes [1]. Management decisions are often shaped by the expertise available at the time of injury and the willingness of patients to engage in extensive and often multiple surgeries following these life-altering injuries [2].

The incidence of TBPI is variable, ranging from 0.69 to 3.9 per 100000 people per year in the USA, 0.58 in the UK, between 0.17 and 0.22 in Japan, 0.2 in the Czech Republic and Slovakia, and 1.75 per 100000 per year in Brazil [3]. These variations reflect local conditions, TBPI service provision, and differing reporting standards. Globally, young males form the predominant demographic, with a pooled prevalence of 93% (95% CI: 90-96%) for males and 7% (95% CI: 4-10%) for females, resulting in a combined male-to-female ratio of 13.3:1 [4]. In Low- and Middle-Income Countries (LMICs) the typical patient is under 35 years of age and involved in trauma related to motor vehicle transport, specifically motorcycles, and suffers closed injuries with dominant limb involvement [4,5]. The individual and societal impact of TBPI is severe with loss of physical function and vocation, lifelong neuropathic pain, and considerable care burden [6]. Late diagnosis and treatment of nerve injuries lead to poorer functional outcomes [7]. The predominant mechanisms of TBPI are traction, rupture, or spinal cord avulsion. Other mechanisms include laceration occurring from displaced fragments of bone following fracture and are known to occur even in closed injuries [8]. The brachial plexus may also be compressed by hematoma, joint dislocation, or fracture displacement resulting in palsy. There are two anatomical types of TBPI: supraclavicular lesions, where the predominant injury occurs at the root or trunk level, and infraclavicular lesions, which affect the divisions, cords, and branches. Severity is assessed based on the nerves involved and the pathophysiological grade of injury to each nerve [8].

Reconstruction algorithms for TBPI have been defined according to anatomical injury pattern, upper brachial plexus roots C56 (+/-C7), lower brachial plexus nerve roots C8-T1, and pan plexopathy. The main objectives of reconstructions to date broadly include shoulder and wrist stability, elbow and digital flexion, and hand sensation. The results of nerve ruptures treated with grafts, however, remain unreliable. Intra-plexal nerve donors provide predictable outcomes when used for transfer, while extra-plexal nerve donors may be considered in more severe cases [9]. Crowe et al. reported geographical variation in the techniques used for shoulder and elbow function in upper TBPI and for reinnervation in pan-plex TBPI. The results of fascicle transfer for elbow flexion demonstrated less variation between regions, reflecting the defined indications for this surgical technique. However, the results of intercostal nerve transfer to the musculocutaneous nerve (ICN-MCN) measured using the British Medical Research Council (BMRC) grade were superior in Asia compared to other regions. Nerve transfers around the shoulder demonstrate more technique variation in the literature and publications from centers in Europe report significantly better outcomes than those from other regions [10].

TBPI management in LMICs may be hindered by delayed presentation, limited availability of centers with experience or expertise in management, and limited resources, resulting in suboptimal outcomes [11,12]. A lack of resources can contribute to publication bias toward centers located in regions with more established healthcare systems [13]. However, the lack of standardized guidelines for the management of TBPI is not specific to LMICs [2]. This scoping review focuses on assessing the current availability of published guidelines for the management of TBPI and considers the format, content, and clinical relevance of these guidelines for international use.

## Review

Materials and methods

**Figure 1:**
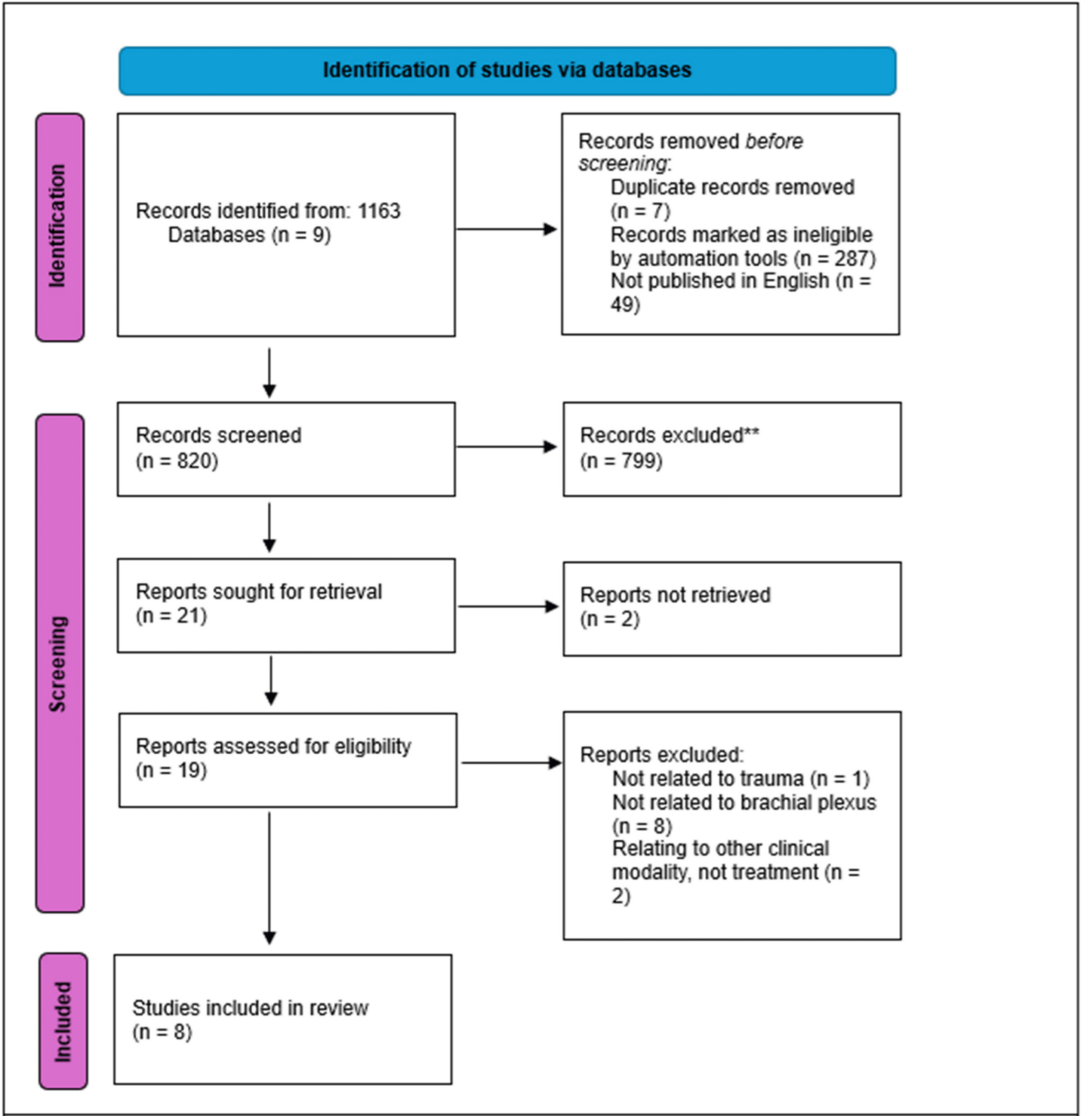
PRISMA outline of literature review on published guidelines for TBPI ^** ^Records were excluded after title screening. Each title was screened in full according to the methods described. Summary of steps used to identify the current literature available for the management of TBPI. Eight papers were deemed relevant as 'guidelines for the management' of TBPI from a total of 19 assessed as eligible out of 820 records screened. Inclusion criteria: Published guidelines, over 18, frameworks for the management of adult BPI, and protocols for managing acute TBPI. Exclusion criteria: Non-English, not mentioning guidelines, rehabilitation regimens, management option discussions, studies on neonates or children, single nerve root TBPI management, and publications before 1990. PRISMA: Preferred Reporting Items for Systematic Reviews and Meta-analyses; TBPI: traumatic brachial plexus injury; BPI: brachial plexus injury

The scoping review was conducted in accordance with the Joanna Briggs Institute methodology for scoping reviews [14]. The Preferred Reporting Items for Systematic Reviews and Meta-analyses extension for scoping reviews (PRISMA-ScR) guidance was used to ensure accurate and objective reporting [15]. A search of English language literature using the terms “traumatic brachial plexus injury” and “guideline” OR “protocol” OR “framework” OR “algorithm” was conducted with expanded Boolean terms and medical subject headings (MeSH). The primary search was conducted in September 2024 by the University Hospitals Birmingham (UHB) Library and Knowledge Service. Databases searched included Embase, Medline, Ovid Medicine, NHS Knowledge, PubMed, BMJ Best Practice, Scopus, Cochrane Library, and Google Scholar. Titles were screened for eligibility (Figure 1) and articles meeting the inclusion criteria were retrieved in full. Article reference lists were reviewed to identify any publications missed by the primary search, which were then included. A guideline was defined as a document advocating specific methods for diagnosis and treatment, describing management, or including an algorithm for the treatment of TBPI.

Study Screening

Two reviewers independently screened titles, abstracts, and full texts of retrieved studies. Discrepancies during the title and abstract screening stages were resolved by automatic inclusion to ensure thoroughness. Discrepancies during full-text screening were resolved through consensus between the reviewers or escalated to the senior author for adjudication.

Selection Criteria and Data Extraction

Articles meeting the criteria included reports of published guidelines on an adult patient population, defined as over 18, frameworks for the management of adult brachial plexus injury (BPI), and protocols for managing acute adult TBPI. Studies that were excluded at screening were those not published in English, as translations may lack accuracy, study’s referencing guidelines for non-TBPI or other BPI modalities (e.g., shoulder dystocia, brachial plexus anesthesia administration, tumor-associated BPI, gun-shot related, sporting, radiation-induced, or obstetric BPI), or studies not mentioning guidelines, studies of rehabilitation regimens, articles on management strategies alone with no defined scope for a guideline, studies on neonates and children or guidelines focusing on a single brachial plexus nerve root management and those published before 1990, as practice would have changed substantially subsequently. The papers were extracted for full-text article analysis as per Cochrane Handbook recommendations. Data items collected included injury patterns, timing to surgery, pre-operative management, neurophysiological testing, imaging, reconstructive priorities, surgical techniques, defined interventions for affected nerves, outcome measures, follow-up, trial (of guidelines) on a patient cohort, and management algorithm.

Data Analysis, Quality Assessment, and Evidence Synthesis

The quality of the identified guidelines was evaluated using a modified version of the Appraisal of Guidelines for Research and Evaluation II-global rating scale (AGREE II-GRS) tool [16]. Out of 22 criteria, 11 were selected for their relevance to this article, the type of guidelines published, and their pertinence in assessing the elements that define the quality of a guideline. These selected criteria encompassed key aspects including the clarity of the guideline's objectives, the specificity of the guideline questions, the identification of an appropriate patient population, the inclusion of relevant professional groups in the guideline development process, the consideration of patients' perspectives, the definition of the target users, and the piloting of the guideline among its intended users.

Results

The initial search yielded 1163 results, Figure 1. After excluding duplicates (n=7), ineligible entries (n=287), and non-English publications (n=49), 820 records underwent title screening with 799 excluded. Abstracts of 21 studies were reviewed and two were excluded due to inaccessibility. Of 19 eligible full-text reports, one lacked trauma-specific guidance, seven were irrelevant to BPIs and two lacked clinical management recommendations. Eight studies met the inclusion criteria and included six original research articles, one national guideline, and one departmental guideline.

Guidelines were collected from Italy, India, the Netherlands, Scotland, Germany, Dubai, and North America. Most guidelines were produced by European institutions and incorporated the management of 130 patients with recorded outcomes, Table 1. In all cases, there was no single comprehensive published guideline (including all elements of management, i.e., nerve transfer guidance) by a national body that incorporated consideration of all areas of practice for the multi-disciplinary team (MDT), indicating the complexity and variability associated with the management of TBPI.

**Table 1:** Summary of literature review and management recommendations from published guidelines Eight guidelines were found following a review of the literature. These were analyzed to determine whether they provided guidance on the timing of surgery, work-up, surgical priorities, specific techniques, management of individual nerves, follow-up guidelines, outcome measures, and whether they were piloted on patient groups [17-23]. NR: not reported; F/u: follow-up; Gang: ganglionic; EDX: electrodiagnostic tests; SSN: suprascapular nerve; SAN: spinal accessory nerve; XI: spinal accessory nerve; ICN: intercostal nerves; TD: thoracodorsal nerve; AN: axillary nerve;  Cons.: conservative; Mx: management; US/USS: ultrasound; MRI: magnetic resonance imaging; DRG: dorsal root ganglion; Ext: external; MSCN: musculocutaneous nerve; MN: median nerve; LTN: long thoracic nerve; Abd: abduction; EDS: electrodynamic stimulation; NAP: nerve action potential; CMAP: compound muscle actin potential; BMRC: British Medical research council; MRC: Medical research council; CXR: chest X-ray, BPI: brachial plexus injury, NSAIDS: non-steroidal anti-inflammatory drugs

Date	Country	Authors	Target audience	Study design	Timing to surgery	Work-up and imaging	Priorities and surgical techniques	Nerves and management	Outcome measures and follow-up	Patient groups	Total sample size
1994	Italy	Ferraresi et al. [17]	Clinicians	Case series	3-6 months	Examination, baseline EMG, CT myelography, and MRI	Intra-op NAP and CMAP testing; extra/intra-plexus neurotization, nerve graft, 10/0 nylon, histological exam intra-op, search for viable stumps	C5-6: intra-plexus neurotization for stretch, direct grafts. C7-T1: surgery restoring wrist+finger flexion. C5-T1: extra-plexus neurotization with SSN/ICN	BMRC grading, 18-month follow-up	Acute traumatic BPI	48
2000	North America	Gutowski and Ornstein [18]	Clinicians	Expert review	Within 6 months	Clinical evaluation, EMG, CT myelography, and CT angiogram if vascular injury is suspected	Restoring M4+ elbow flexion, but requires stability of the shoulder to achieve. Then, wrist and finger extension	C5-6 rupture or traction: primary neurorrhaphy, then muscle transfer; if avulsion: ICN & C7 nerve root. SSN and phrenic for shoulder stabilization. If >30-40: muscle transfer	BMRC grading, no length of time for f/u described	Acute traumatic BPI	NR
2016	India	Sinha Sumit et al. [19]	Clinicians	Expert review	Pre-gang: immediate. Post-gang: 3-6 months	EMG every 3-4 weeks. No guidance on neuroimaging	Restore shoulder stability/ abduction, elbow flexion, median nerve function, finger flexion, and grasp	C5-6: stabilize shoulder, SSN to SAN, shoulder abduction, Somsak/TD to AN, elbow flexion, Oberlin. C5-7: Pre-shoulder ICN to AN. C8-T1: Pre-gang nerve transfers, post-gang nerve grafts to cords	Consult physiotherapy, no f/u regimen specified	Post-acute TBPI	NR
2018	Leiden, Netherlands	Pondaag [20]	Clinicians	Case series	2 weeks to 2 months	ABC assessment, examination, EMG: (1) MRI plexus, (2) CT myelography, (3) diffusion tensor imaging, (4) high-frequency US	Restoring elbow flexion is a priority. Identifying the site of resection between damaged and viable tissue is essential. Management of CSF leakage involves plugging and neurosurgical intervention	Displaced, ruptured, or avulsed nerves are repositioned proximally, direct intra-plexus coaptation, DRG dissected, and post-DRG excision and grafting. Grafting >transfer	MRC grade examination, F/u over 26 months	Acute TBPI	36
2020	Scotland	NHS Scotland [13]	MDT	Published guideline	NR	Resuscitate, examination, muscle testing, sensation testing EMG. CXR, MRI C-spine, and CT myelography	Assessment to rule out vascular injury, then referral to brachial plexus service. Formal brachial plexus injury. Improve motor/sensory function, mainly repair elbow flexion	Primary repairs: nerve grafts/reconstruction, nerve transfers; Secondary: bony fusions, tendon transfers	DASH score, HAD score, Narakas score, clinical and physiotherapy assessment, passive movement program. No length of follow-up specified	Acute TBPI	NR
2020	Germany	Tiefenboeck et al. [21]	Clinicians	Case series	NR	History, examination, X-ray in two planes, arthro MRI, MRI, and CT. If BPI: Plexus MRI and EMG evaluation	Pre-op mx: vitamin B prescription, physio, splinting/immobilization, limb elevation, lymphatic drainage	Neuro examination and imaging, referral to plastic surgery team for urgent surgery if required	7 months (range of 1-18 months) regimen including physio, electrotherapy, and lymph drainage	BPI following shoulder dislocation, pre-op	46
2023	Dubai	Debora Garozzo [22]	Clinicians	Expert review	Avulsion: immediate; Post-gang: after 4-6 months	History, examination, physiotherapy; 3D MRI, MR myelography, 3D MRI after 1 month, angiography, 2D CT for scapula reconstruction	Reinnervate spinatus muscles/deltoid/ biceps; regain elbow flexion, shoulder stabilization, and abduction. Two phases: exploration and micro-reconstruction with 9-0 non-absorbable monofilament, microscope, intra-op EDS stimulation, and GA explorations	C5-C7: Spinatus: XI to SSN; Biceps: medial cord to MSCN; Deltoid: TD to AN via graft. C8-T1: Brachialis of MSCN to MN. Pan-plexus: XI-SSN and graft to the anterior upper trunk. Pan avulsion: XI-SSN (T3-T6) or T4-T6 to MSCN	BMRC evaluation, Tinel’s evaluation, Immobilize for 2 weeks. Physio afterward. No length of f/u specified	Post-acute TBPI	NR
2023	North America	Karkare/Complete Orthopedics [23]	MDT	Published guideline	NR	History, exam, EDX MRI, or myelography, CXR	-	Conservative mx options: NSAIDS, opioids, anti-depressants, anti-convulsant, rest, and gentle physio	Physio with periods of rest from 4-10 weeks, depending on nerve injured. No length of f/u specified	Post-acute TBPI	NR

Structure and Quality of Guidelines

Seven of the eight guidelines reviewed described BPI, injury patterns, and patient demographics, with six containing treatment algorithms [13,18-22]. Four followed a standardized scientific journal structure, while two focused on surgical aims and approaches [18,20-22]. The NHS Scottish National TBPI service manual addressed injury categorization, surgical and physiotherapy options, and rehabilitation goals [13]. In contrast, Karkare's non-peer-reviewed web article outlined nerve-specific history, diagnostics, and management, offering limited management advice for TBPI care [23]. A modified AGREE II-GRS analysis, Table 2, identified inconsistency in the description of questions to answer in guidelines, encompassing all relevant professional groups, piloting management among patients, and reflecting patients' views [16]. There were more positive outcomes for defining target users of guidelines and identifying appropriate patient populations for the use of guidelines.

**Table 2:** Modified AGREE II-GRS analysis of eligible guidelines for the management of TBPI Modified criteria relevant to the analysis of these clinical guidelines were selected and applied to the guidelines identified in the literature review. This analysis found that the main areas of consensus between the guidelines were "target users of the guideline defined" and "appropriate patient population identified" [17-23]. TBPI: traumatic brachial plexus injury; AGREE II-GRS: Appraisal of Guidelines for Research and Evaluation II-global rating scale

	Ferraresi et al. (1994) [17]	Gutkowski and Orenstein (2000) [18]	Sinha et al. (2016) [19]	Pondaag et al. (2018) [20]	NHS Scotland (2020) [13]	Tiefenboeck et al. (2020) [21]	Garozzo (2023) [22]	Karkare (2023) [23]
Were the objectives of the guideline described?	Y	N	Y	N	Y	Y	Y	Y
Were specifically described questions to be answered in the guidelines?	Y	Y	N	Y	N	Y	N	N
Is the appropriate patient population identified?	Y	Y	Y	Y	Y	Y	Y	Y
Does the guideline development group encompass relevant professional groups?	Y	Y	N	Y	Y	Y	Y	N
Are patients' views reflected?	Y	N	N	Y	N	N	N	N
Are the target users of the guideline defined?	Y	Y	Y	Y	Y	Y	Y	Y
Guideline piloted amongst target users?	Y	N	N	Y	Y	Y	N	N
Was the guideline process of creation transparent and reproducible?	N	N	N	Y	Y	N	N	N
How complete was the information to inform decision-making?	Y	N	N	Y	N	N	Y	N
Are the recommendations clinically sound?	Y	Y	N	Y	Y	N	Y	N
Are the recommendations appropriate for the intended patients?	Y	Y	N	Y	Y	Y	Y	Y

Timing to Surgery

Five of the articles mentioned a definitive timeline for surgery, using CT myelography and/or MRI to differentiate the type of injury as either pre- or post-ganglionic [17-20,22]. Three advised emergent nerve transfer surgery following avulsion or pre-ganglionic injury as delay could prevent meaningful recovery [20]. However, other authors suggested that post-ganglionic injuries should be observed for two to three months to determine the extent of functional recovery before surgery. If an open injury occurred, urgent surgery was required, along with any subsequent reinnervation within one year of the injury. One author advocated for brachial plexus exploration within two weeks of injury [20]. They hypothesized a greater likelihood of achieving direct end-to-end neurorrhaphy of ruptured nerve stumps, with a reduced likelihood of requiring nerve grafts due to the absence of scar tissue, better tissue pliability, and the closer proximity of injured nerves. However, this approach carries risks, including the disruption of recovery for partially injured nerves and the difficulty of assessing anatomy in the immediate aftermath [20].

Surgical Priorities and Adjunct Procedures

Five of the guidelines stated clear objectives for surgical priority setting and achieving functional targets, including restoration of shoulder stabilization, elbow flexion, and hand function [8,17-19,22]. The importance of these priorities and the recommended sequence of reconstruction were absent in all guidelines. Intra-operative evaluation of nerve viability was not specifically mentioned in most reports. However, methods suggested for use included microscope assessment of in-continuity lesions, intra-operative nerve stimulation, and fresh frozen staining of donor and recipient stumps, although most authors indicated a preference for macroscopic visual inspection [17,19,22]. The merits of supra- and infra-clavicular exploration were mentioned by one author but were not common across the board. Broadly, the operative approach and visualization were not specified by the majority of authors [22]. The clarity of operative details, including magnification, sutures, adjuncts for coaptation, and patient positioning, was not universally disclosed. Two articles focused on utilizing multiple nerve transfer options, as opposed to a distinct algorithm-based pathway [19,22]. 

Surgical Techniques and Targeted Plexus Reinnervation

Targeted reinnervation for plexus reconstruction was based on the nerve roots affected, four of the eight authors described formal management options for each category of affected nerve roots [17,19-20,22]. Only one article offered a systematic overview and attempted an ordered approach for reconstruction, but did not state a categorical order for nerve transfers or clearly define indications [22]. Five of the guidelines described the need to consider intra- and extra-plexal donors for reconstruction [13,17-19,22]. Procedures recommended for reconstruction were considered for C5-6 +/-7, C8-T1 and pan plexopathy (C5-T1) affected nerve roots.

The two, C5-6 root injury, targets commonly reported were stabilizing the shoulder and restoring elbow flexion. Suggested options for reinstating shoulder function included spinal accessory nerve (SAN) to suprascapular nerve (SSN) transfer, triceps branches to the axillary nerve (AN), or thoracodorsal nerve to AN. For elbow flexion, the medial pectoral nerve to musculocutaneous nerve transfer or the double Oberlin transfer were commonly suggested [17,19,22]. The objectives for C8-T1 injury reconstruction were restoring finger flexion and grasp function. For pre-ganglionic injuries, the main aim was restoring median nerve function from unaffected nerve roots, i.e., supinator to AIN and FDS to FPL. For radial nerve function transfer, options included a brachioradialis or supinator to the posterior interosseous nerve [19,22]. For post-ganglionic injuries, ipsilateral or contralateral C7 to the lower trunk or medial cord direct neurotization, with or without interposition grafts, was suggested [19]. For pan-plexal (C5-T1) injuries, the authors agreed that reconstructive options are limited and that a multi-staged, extra-plexal donor approach could be considered in the reconstructive algorithm. Intercostal nerves from T3-6 and contralateral C7 coaptation have been suggested for the reconstruction of shoulder abduction and elbow flexion; however, these procedures are complex, and the current reconstruction strategies reported have offered limited functional gains to date [19,22].

Measurement of Outcomes and Follow-up

Six guidelines addressed functional outcome measures and detailed follow-up protocols [13,17-23]. Heterogeneous use of patient-reported outcome measures (PROMs) limited comparative analysis. Regular follow-up was emphasized inconsistently across the articles reviewed for complication monitoring, recovery assessment, and rehabilitation optimization. However, the lack of standardized outcome measures complicated evaluation of surgical algorithm efficacy.

A summary table explaining the analysis of each guideline’s applicability is presented in Table 3.

**Table 3:** Overview of published TBPI guidelines: general analysis A critical analysis of each guideline’s main strengths and limitations, along with points for consideration about the article, will be documented to give some context, according to the authors. TBPI: traumatic brachial plexus injury; PROMS: patient-reported outcome measures; MDT: multi-disciplinary team

Author	Strengths	Limitations	Points for consideration
Ferraresi et al. (1994) [17]	A clear outline of patient assessment, an overview of follow-up, nerve transfer strategy rationalized, and reporting of outcomes measures or PROMS	No definitive treatment algorithm, no prioritization of nerve transfer options, specific to surgeons only, not other members of MDT, and single-center study	Written at the time of the first Oberlin transfer report. Single-center study. Well-written, possibly only missing an algorithm. Contains a reasonable level of information for determining a nerve transfer strategy
Gutowski and Orenstein (2000) [18]	Clarification of assessment and investigation of patients, clear indications for procedures, nerve transfer strategy rationalized, an algorithm for emergency management, and some wider relevance to allied healthcare team	Related to only C5-6 injuries, not other guidelines; reports guidelines, but not in title; no inclusion of allied health care team; no reporting of outcomes measures or PROMS; no reporting of patient cohort outcomes	Very early guideline, focused on the management of trauma and C5-6. Useful in determining emergency management for TBPI, with a clear indication of the fundamental reasons for surgery. Lacks information on strategies outside of C5 and C6, as well as outcome measures
Sinha et al. (2016) [19]	Broad coverage of nerve transfer strategies, algorithm reporting of transfers, rationalizing strategies for nerve transfers, paper meets aims and objectives set out in methodology	No reporting of outcomes measures or PROMS, no definitive follow-up regimen, no definitive stepwise strategy for nerve transfers, specific to surgeons only, and not other members of MDT	Report based on experience gained in a large center in India, although no patient cohort or outcomes are described. No sequence of transfers is described. Hence, it is useful in identifying options, but not in determining which ones to use and when
Pondaag et al. (2018) [20]	Clarification of assessment and investigation of patients, algorithm of determining early vs. late surgery, case series of patients included for discussion of results, and PROMS measurement of outcomes	Does not distinguish which procedures are more amenable to early vs. late surgery, rationales for procedures based on departmental procedures, minimal discussion of alternative nerve transfer surgeries, single surgeon, and single-center	Clinically experienced authors included a case series for the evaluation of management. A concise paper that explains the rationale for nerve transfer management well. Useful in determining clinical judgment
NHS Scotland (2020) [13]	Clear outline of management through all parts of patient TBPI care, definitive follow-up regimen reported, clear guidance on referral criteria for specialist treatment, provides some outline of nerve transfers, covers breadth of TBPI better than rest, and contains readily available/useable assessment forms and triage guidance	Written specifically for an allied healthcare specialty team, not able to assist in education on nerve transfers or in detailing strategy, and no reporting of outcome measures or PROMS	Designed for the allied healthcare team with a patient focus and easy to read. Not designed to guide clinical decision-making for nerve transfers, but contains a wide breadth of information useful for educating a spectrum of healthcare professionals
Tiefenboeck et al. (2020) [21]	Standard format of the article, reasonable overview of nerve lesions and follow-up, clear algorithm of triage according to nerve injury and investigation, and advice on the management of complete and incomplete nerve lesions	Focuses more on triage than strategies for nerve repair, no recruited patients required surgical management, not useable for determining nerve transfer strategy, and no reporting of outcomes measures or PROMS	Did not recruit any patients who required surgical management, so it is hard to know if the algorithm for assessment works for those requiring surgical management
Garozzo (2023) [22]	Most comprehensive published guideline containing management for most TBPI scenarios, a clear rationale for each procedure documented, caveats for clinical decision making and practice included, the full breadth of management from initial assessment to follow-up documented, and most complete of all the articles reviewed	Limited reporting of outcomes measures or PROMS, no report of the patient population managed with these guidelines, no referenced measures for nerve transfer suggestions, no expert opinion level evidence, specific to surgeons only, and not other members of MDT	Clinically experienced authors with a breadth of experience and understanding of surgical strategies. Quite specific to surgeons, with a good explanation of the rationale for nerve transfer strategies. The most surgically comprehensive of the guidelines evaluated
Karkare (2023) [23]	Clear format for detailing nerve lesions and treatment, covers the breadth of nerve injuries, a system for triage of nerve injuries, clear objectives from the outset, emergency management outlined, and provides caveats: not for clinical decision-making	Focuses more on triage than strategies for nerve repair, not useable for determining nerve transfer strategy, no reporting of outcomes measures or PROMS, and no inclusion of allied health care team	Largely a document provided for the purpose of navigating medical litigation. As a guideline, it covers most workplace nerve injuries and emergent management but does not go beyond this or offer much clinical direction

Discussion

This review found eight relevant guidelines from a search of nine databases. The guidelines identified lacked consistency in structure and had limited breadth, not describing sequential management for all stages of TBPI. Each guideline tended to focus on specific areas, with some offering surgical management guidance, while others focused on other elements of care, including rehabilitation or basic treatment for medicolegal evaluation. Across all guidelines, there was a lack of consistency in outcome measurement for TBPI and no standardized description of outcome reporting tool use. The modified AGREE II-GRS analysis did find that the published guidelines were able to define appropriate target users and identify patient populations to which the suggested management was relevant.

Differences in Practice and Model for Future Development

Inherent in the disparity of suggested management quality is a difference in training and clinical experience. Work by Belzberg et al. (2004) demonstrated differences in the opinion of BPI management through an international survey of peripheral nerve surgeons, focusing on choices for nerve transfers, the management of neuroma-in-continuity, and diagnostic approaches to defining injury [24]. This was also reflected in later work by Lubelski et al. (2023), who surveyed international peripheral nerve surgeons and found persistent differences in preferences for imaging modalities and choices of nerve transfers in line with scientific developments, although recent consensus was found on some reconstructive targets and goals [25]. If such variations in clinical practice exist among experts from countries with established specialist services, particularly concerning fundamental aspects of management like imaging and the selection of nerve transfers for common presentations, it is unsurprising that nations with limited resources and expertise may face challenges in initiating and optimizing TBPI care.

Additionally, the availability of TBPI expertise will vary according to resources and patient numbers, with some areas experiencing low demand for peripheral nerve surgery (PNS) and a lack of service infrastructure. As a result, the medical staff serving these populations have decreased exposure and technical knowledge, reducing the efficacy of TBPI care provision [12]. However, where there is demand, expertise is required, and support for developing a staff of trained medical practitioners to provide an effective service is essential, along with clearly defined minimum standards of practice to underpin this.

Exposure to training in the management of TBPI is known to vary between specialties, with one recent review highlighting the disparity in peripheral nerve procedure exposure between general and neurosurgical trainees and their orthopedic and plastic surgery counterparts [26]. If this level of scarcity of experience can exist within a well-resourced educational system, one can imagine the paucity of educational experience that pervades resource-constrained settings.

Initiatives have begun to find a model for implementing training and service development to address this issue. A working model of integrated local and international health collaboration in Serbia has led to the establishment of a fully integrated PNS service with the aid of the World Federation of Neurosurgical Societies [27]. A one-size-fits-all model may not work at present, and other nations may face different barriers to entry in replicating such endeavors. However, this offers an internationally recognized template that could be used for service development in the future within resource-limited settings.

More Than Just an Algorithm

Introducing the systematic change required to grow and develop a TBPI service requires planning and engagement from key stakeholders. Designing a set of measures to increase the standards of TBPI care could borrow several ideas from high-income countries (HICs), of which the most appropriate and easily adaptable could include establishing a global consortium for TBPI care, creating a research group within such a consortium to determine best practices and resource allocation, publishing a guideline on the management of TBPI, developing an online educational training portal with a defined curriculum for all MDT staff, creating a portable trans-territory simulation training course in nerve transfers, establishing a core outcomes database, and fostering links between HIC and LMIC healthcare systems [28,29]. All measures are anticipated to address some of the gaps that persist in knowledge, practice, and outcomes for TBPI patients between HIC and LMIC health systems.

On a practical basis, establishing a global consortium would provide oversight for service and educational development and serve as a communication hub between HIC and LMIC healthcare systems. However, realizing such a consortium presents challenges, including securing an institutional base, achieving stakeholder buy-in from government levels and regional health planning bodies, as well as sourcing adequate funding for key provisions such as theater space, specialist equipment, teaching materials, and technological infrastructure to support service delivery [27]. Central to the consortium’s mission would be a dedicated research team comprising clinicians, bioinformatics experts, health managers, and service-commissioning bodies with experience in the design, management, and scaling of health initiatives. This team would analyze clinical data from newly established services to identify effective, resource-rationalized, and geographically context-appropriate interventions. Collaboration with HIC healthcare units experienced in TBPI management could provide additional expertise in research and oversight of applications. However, the challenge of identifying and recruiting this network of talent to engage in such a service would form a substantial barrier to this initiative.

Institutional reform on this scale requires a foundation of evidence-based outcome measures. One potential solution is the establishment of a centralized database to record patient utilization of services, alongside both clinical and patient-reported outcomes [30]. Existing core outcome measures for the TBPI patient cohort could be adapted and incorporated into such a database, providing a standardized framework for data collection and analysis [29]. Challenges to implementing this approach may include costs associated with database access, including equipment, setup, staff training, and internet access, as well as expenses for data management, promotion, and, ultimately, the application of findings into clinical practice. However, the benefits of such a database are substantial. It would enable rapid meta-analyses of clinical trends and facilitate multi-center collaboration, supporting the development of geographically relevant nerve transfer strategies and treatments in the absence of a definitive standard of care [30]. To ensure its effective use, the database should incorporate safeguards for data input, appropriate utilization, and dissemination of information. Secure access controls and robust oversight mechanisms would also be key [30]. Finally, the database’s utility would depend on the quality of the data captured, necessitating comprehensive training for all users, supported by appropriate coordination and oversight. Despite the initial barriers, establishing a centralized database represents a foundational step toward understanding current gaps in TBPI management, improving clinical outcomes, and informing the development of future services [29].

Developing targeted, multidisciplinary guidelines with input from national health bodies and a global health-focused consortium would provide a standardized framework for TBPI management [27]. These guidelines should focus on defining minimum treatment standards, achieving consensus on nerve transfer regimens for specific presentations, and outlining the resources required for treatment implementation. Such efforts would enhance practice across diverse geographical settings and improve patient outcomes. Educating a workforce capable of delivering specialized TBPI care would be a foundational pillar for this service [11,12]. International efforts are underway to create accessible and scalable online educational platforms that could facilitate this goal, such as Canada’s Ptolemy educational initiative, which has demonstrated positive impacts on surgical training and technique dissemination [28]. Simulated surgical training environments may offer opportunities to enhance technical performance and tailor treatment strategies to specific resource and contextual needs [26]. Barriers to workforce education around this might include limitations in training models, costs of in-person workshops, and the logistical challenges of organizing such events. Overcoming these challenges through innovative training methods, lowering barriers to access, and raising awareness is essential for building capacity in TBPI care delivery [28].

The systematic development of TBPI services requires coordinated international efforts to address gaps in care delivery and outcomes. Establishing a global consortium centered around TBPI treatment expertise, creating a core outcomes database, publishing standardized guidelines, and investing in workforce education are critical steps toward achieving equitable and effective TBPI management across HIC and LMIC settings.

Considerations for Limited Resource Settings

Currently, no standardized international guidelines exist for the management of TBPI in resource-limited settings. Timely recognition and intervention are critical in environments where care is influenced by the local health economy, available equipment, timing of presentation, surgical expertise, and limited rehabilitation resources [28]. Centralizing care to select geographical areas may begin to address these high-complexity and low-incidence injuries, but it could further limit access to optimized care, leaving a patient cohort who may remain underserved. Advanced reconstruction techniques used in developed countries may not be directly applicable to LMICs due to resource and skill limitations; however, carefully informed application of elements of these treatments may be offered only where the expertise exists to facilitate this. Any future guidelines must consider the appropriateness and feasibility of treatment algorithms within limited-resource settings to improve equality in patient outcomes.

Limitations of This Review

This review evaluated the availability and components of existing guidelines for TBPI management without advocating for specific algorithms or content and was therefore limited to published and accessible guidelines. Comparative assessment was hindered by the heterogeneity of focus across guidelines, with some emphasizing reconstruction over feasibility or rehabilitation outcomes, and often lacking standardized reporting of outcomes, which introduces the risk of bias. Advances in TBPI management have rendered retrospective reviews potentially outdated, as they reflect the prevailing knowledge at the time of publication, not the latest developments and their application. There is a lack of RCT data on effective management strategies, with no accounting for concomitant nerve releases in TBPI cases. Similarly, some guidelines may not have been published but are actively in use at regional and local centers, which we did not have access to, and the guidelines we reviewed may not reflect real-world practice. It is possible that the limitation of language restrictions in the search may have biased the findings.

## Conclusions

Standardizing TBPI care in LMIC settings remains a significant challenge due to the lack of consensus on surgical management and outcome reporting. While pre-ganglionic injuries universally require emergent management, controversy persists regarding the timing of surgery for post-ganglionic injuries, with no definitive protocol established to date. Although well-documented nerve transfer targets exist for C5-C6 lesions, there is little agreement on optimal targets for C8-T1 injuries and pan-plexus avulsions, with limited clinical success reported in the latter. This highlights the ongoing uncertainty in TBPI management, the difficulty in defining the best contextual nerve transfer algorithm, and underscores the need for a standardized guideline tailored to LMICs to ensure consistent and effective care.

Addressing this gap requires the formation of a global consortium to oversee TBPI care, along with a centralized outcomes database to facilitate comparative analysis and inform best practices. Developing an adaptable, evidence-based guideline specific to diverse LMIC settings is crucial and would require support from scalable workforce education initiatives, such as online remote learning courses and simulation-based training programs. Despite challenges related to funding, infrastructure, and key stakeholder coordination, these initial endeavors represent vital investments toward equitable and effective care for this at-risk patient group. Future efforts should integrate both local and international expertise, prioritizing resource-appropriate interventions to enhance patient outcomes across different healthcare settings.
